# Error in Author Affiliations

**DOI:** 10.1001/jamanetworkopen.2026.44193

**Published:** 2026-10-20

In the Original Investigation titled “Large Language Models and Adverse Event Detection Within Immunotherapy Clinical Trials,”^1^ published September 3, 2026, author Julian C. Hong, MD, MS, should have also had affiliations listed for the Helen Diller Family Comprehensive Cancer Center, University of California, San Francisco, and Weill Cancer Hub West, Stanford University – University of California, San Francisco. This article has been corrected.^1^
